# KNOX1 Transcription Factors in Plants with a Special Focus on Horticultural Crops: A Review

**DOI:** 10.3390/plants15142127

**Published:** 2026-07-09

**Authors:** Xiaobei Cai, Kehang Chen, Lili Ye, Laiba Bibi, Jingshi Zhang, Tianxin Feng, Cheng Zhang, Yudan Wang

**Affiliations:** 1 Innovation Center for Cell Signal Transduction and Synthetic Biology, Guangdong Provincial Key Laboratory of Plant Adaptation and Molecular Design, Guangzhou University Branch Center of National Key Laboratory of Non-Food Biomass Energy Technology, School of Life Sciences, Guangzhou University, Guangzhou 510006, China32314100015@e.gzhu.edu.cn (K.C.); 32314130069@e.gzhu.edu.cn (L.Y.); 32414100061@e.gzhu.edu.cn (J.Z.); 32414100082@e.gzhu.edu.cn (T.F.); 2 Department of Botany, Centre for Plant Sciences and Biodiversity, University of Swat, Mingora 19201, Pakistan; laibakhan22@163.com

**Keywords:** *KNOX1* gene family, developmental biology, regulatory network, molecular breeding

## Abstract

Class I KNOX1 (KNOTTED1-like homeobox 1) transcription factors integrate gibberellin (GA), cytokinin (CK), and auxin (IAA) signaling to maintain shoot apical meristem identity and coordinate plant organogenesis. This review examines the structural conservation, evolutionary dynamics, and regulatory architecture of *KNOX1* genes across horticultural crops, drawing essential mechanistic context from model species. We synthesize *KNOX1* functions in six agronomic domains, including plant architecture and branching, leaf morphogenesis and ornamental traits, floral development and sex determination, fruit formation and quality, storage organ specification, and abiotic stress resilience. Particular attention is given to recent breakthroughs in cucurbit inferior ovary development, tomato chloroplast patterning, and potato tuber morphogenesis. We identify critical bottlenecks constraining translation, including fragmented regulatory networks, recalcitrant transformation systems in woody perennials, uneven taxonomic coverage favoring annual vegetables over ornamentals and medicinal species, and a near-complete absence of multi-environment field validation. We propose four strategic priorities to bridge this gap: (i) construction of spatiotemporal expression atlases using single-cell and spatial transcriptomics; (ii) tissue-specific and promoter-engineered CRISPR/Cas9 editing to circumvent pleiotropic penalties; (iii) cross-species comparative evo–devo analysis of lineage-specific innovations (compound leaves, inferior ovaries, tubers); and (iv) integrated field trials assessing genotype-by-environment interactions and trait stability. This framework aims to accelerate KNOX1-directed molecular design breeding in horticultural crops.

## 1. Introduction

The KNOX (KNOTTED1-like homeobox) transcription factor family comprises a distinct subfamily of the TALE (Three Amino Acid Loop Extension) superfamily and ranks among the most evolutionarily conserved gene families in plants [1]. The founding member, *Kn1* (*Knotted1*), was identified in maize (*Zea mays*) through loss-of-function mutations that produce ectopic nodular protrusions on leaves, a landmark phenotype that established the framework for all subsequent *KNOX* gene characterizations [2]. As research on homeobox genes accelerated, the *KNOX* family emerged as a core regulatory module within this superfamily, with its members, evolutionary dynamics, and multifaceted biological functions now extensively documented.

The *KNOX* gene family is broadly distributed across the plant kingdom and comprises three subfamilies, *KNOX1*, *KNOX2*, and *KNATM*, distinguished by sequence similarity and expression patterns. The *KNOX1* subfamily comprises numerous plant homologous genes, among which *SHOOT MERISTEMLESS*(*STM*), *BREVIPEDICELLUS*(*BP*)/*KNAT1*, *KNAT2*, and *KNAT6* are four core genes systematically identified in *Arabidopsis thaliana* [3]. *STM* is essential for SAM initiation and maintenance. *KNAT1* and *KNAT6* regulate SAM function, organ boundary specification, and inflorescence architecture. *KNAT2* governs SAM activity and organ separation while transducing ethylene (ET) and cytokinin (CK) signals; its misexpression causes ovule homeotic transformations and outer integument defects, confirming its role in reproductive development [4,5].

Horticultural crops including vegetables, fruit trees, ornamentals, and specialty economic crops, underpin agricultural supply chains, quality of life, and industry modernization. The sector is transitioning toward higher quality, efficiency, diversification, and full mechanization, making plant architecture optimization, fruit quality improvement, ornamental trait innovation, and stress tolerance enhancement principal breeding objectives [6]. Conventional breeding, reliant on phenotypic selection, is constrained by prolonged cycles, low selection efficiency, and difficulty in achieving multi-trait gains simultaneously. These limitations render it inadequate for current industrial demands. Identifying the core genes governing key agronomic traits and dissecting their molecular regulatory networks are therefore essential to transcend conventional breeding constraints and realize molecular design breeding in horticultural crops.

Unlike model plants such as *Arabidopsis thaliana* and *Oryza sativa*, horticultural crops exhibit exceptional morphological diversity and distinctive developmental programs that require independent functional investigation. Within the *KNOX* gene family, the *KNOX1* subfamily serves as the principal regulatory module of plant morphogenesis, displaying marked species specificity and functional versatility in horticultural species. This positions *KNOX1* as a prime target for improving core agronomic traits. *KNOX1* genes regulate internode elongation and architecture in peach (*Prunus persica*) [7], leaf morphology in pineapple (*Ananas comosus*) [8], and storage organ development in potato (*Solanum tuberosum*) [9]. Most recently, *KNOX1* genes in cucurbits were shown to coordinate inferior ovary development and floral sex determination, a finding that revises our understanding of developmental evolution in this crop group [10]. Together, these functions establish *KNOX1* as a central node connecting developmental biology to horticultural breeding.

Research on the *KNOX* gene family has focused predominantly on model plants, and no systematic review has addressed the *KNOX1* subfamily specifically in horticultural crops. An integrated framework connecting gene structure, regulatory networks, trait functions, and breeding applications is lacking. This review synthesizes *KNOX1* structural characteristics, evolutionary conservation, and molecular regulatory architecture; consolidates current knowledge of their roles in plant architecture, leaf morphology, floral development, fruit formation, storage organ specification, and stress tolerance; examines current research bottlenecks; and identifies future directions enabled by multi-omics platforms and precision gene editing. Our goal is to provide a reference framework for *KNOX1* functional characterization and molecular breeding in horticultural crops.

## 2. Basic Characteristics and Evolutionary Conservation of KNOX1 Genes

### 2.1. Gene and Protein Structure

Across horticultural crops, *KNOX1* genes typically comprise 4–6 exons interrupted by 3–5 introns [11,12,13,14,15]. Splice-site architecture is strictly conserved, featuring canonical GT–AG dinucleotides at the 5′ and 3′ boundaries [16,17]. The mapping of functional domains to exons is invariant: exon 1 encodes the KNOX1 and KNOX2 subdomains, exon 2 the ELK domain, and exons 3–5 jointly encode the TALE homeodomain (HD) [4,18,19].

At the protein level, the KNOX1 fold is built almost entirely from α-helices. The KNOX1 and KNOX2 subdomains each form distinct α-helical segments joined by a flexible linker to constitute the MEINOX domain, which mediates heterodimerization with BELL (BEL1-like homeobox) transcription factors [3]. The ELK domain, a compact α-helix adjacent to the MEINOX domain, completes a surface required for nuclear localization and protein–protein interaction [5]. The homeodomain (HD) forms a canonical three-helix bundle. A loop between helices I and II harbors the PYP tripeptide extension, the defining motif of the TALE superfamily [1]. Helices II and III assemble into a helix-turn-helix (HTH) motif that mediates sequence-specific DNA recognition [19,20].

### 2.2. Genome-Wide Identification of KNOX1 Family in Horticultural Crops

Genome-wide identification and systematic characterization of the *KNOX1* family have been completed across a broad range of horticultural crops, including apple, grape, citrus, pineapple, tomato, potato, Toona sinensis, pepper, tea plant, Japanese apricot, orchid, and water lily. These surveys reveal conserved patterns in gene duplication, chromosomal distribution, and promoter cis-element architecture. An ancestral *KNOX* gene duplicated in early land plants to generate the *KNOX1* and *KNOX2* lineages, and subsequent whole-genome duplication (WGD) and segmental duplication have since driven *KNOX1* expansion [21]. In dicotyledonous species such as grape and citrus, segmental duplication is the predominant mechanism of *KNOX1* proliferation [22,23]. Because WGD can give rise to large-scale segmental duplications, both processes have jointly increased *KNOX1* copy numbers across horticultural crops, providing the genetic substrate for functional diversification. Chromosomal analyses further show that *KNOX1* genes are dispersed across multiple chromosomes and rarely co-localize with other *KNOX* family members, consistent with the evolutionary decoupling of functional paralogs [24,25].

Promoter analyses of characterized *KNOX1* genes in horticultural crops consistently reveal enrichment of cis-acting elements responsive to major phytohormones—auxin (IAA), gibberellin (GA), and abscisic acid (ABA)—as well as to abiotic stresses including drought, low temperature, and salinity. Species-specific elements governing light signaling, salicylic acid response, and pathogen defense are also embedded within these promoters [26], implicating *KNOX1* genes as integrative regulators at the nexus of stress adaptation, hormone signaling, and development. Beyond these shared regulatory themes, *KNOX1* genes show pronounced species-specific divergence in copy number and expression profile. Copy number is tightly constrained: grape and citrus maintain 4–6 *KNOX1* copies [22,23], whereas the monocots pineapple and orchid retain only 3–4 [8,12], a disparity that likely reflects the distinct selective pressures imposed by organ-specific developmental programs. Expression profiles display equally compelling tissue specificity, a disparity likely reflecting distinct selective pressures imposed by organ-specific developmental programs. In pineapple, *AcKNOX1* is predominantly expressed in stems [8]; in apple, *MdKNOX1* accumulates preferentially in flowers and fruits [26]; and in orchid, *PeKNOX1* is sharply enriched in meristematic regions [12]. These divergent patterns underscore the capacity of *KNOX1* genes to acquire specialized regulatory functions tailored to the unique developmental demands of each species.

### 2.3. Evolutionary Characteristics

*KNOX1* genes have retained extraordinarily conserved structural features throughout plant evolution. All four principal domains, KNOX1, KNOX2, ELK, and HD, exhibit stringent sequence conservation, with the KNOX1 domain achieving near-absolute invariance across all 22 class I sequences examined, registering an E-value of 4.3 × 10^−309^ [27], a degree of conservation that approaches the theoretical limit. At the functional level, the shoot apical meristem (SAM) maintenance pathway governed by *KNOX1* genes displays equally remarkable evolutionary conservation spanning monocotyledonous and dicotyledonous lineages [28]. Whether in monocotyledonous species such as pineapple and orchid, or dicotyledonous species such as tomato and potato, *KNOX1* fulfills an indispensable and irreplaceable role in sustaining apical growth and organogenesis by enforcing the suppression of cell differentiation and perpetuating meristem stem cell identity [29]. This core regulatory logic, suppression of differentiation coupled with maintenance of stem cell potency, constitutes a deeply entrenched developmental paradigm that has been faithfully preserved across hundreds of millions of years of angiosperm diversification.

Beyond their deep evolutionary conservation, *KNOX1* genes have undergone profound functional innovation within horticultural crops, serving as primary architects of species-specific morphological diversification. In compound-leaved horticultural species such as tomato and strawberry, *KNOX1* genes orchestrate leaf margin dissection and leaflet initiation through precisely calibrated spatiotemporal expression dynamics constituting the decisive regulatory switch that differentiates compound leaf morphology from simple leaf architecture [30]. In inferior ovary-bearing crops such as apple and pear, *KNOX1* drives the fusion and coordinated growth of the receptacle and ovary, functioning as the central regulatory determinant governing fruit morphogenesis and the formation of edible tissues [26]. In tuber-bearing crops such as potato and yam, *KNOX1* genes command storage organ morphogenesis by simultaneously suppressing stolon elongation and activating starch biosynthetic pathways [9]. Collectively, these findings reveal that *KNOX1* genes have been repeatedly and independently co-opted across horticultural lineages to sculpt the very traits that define the agronomic and commercial value of horticultural crops— leaf complexity, fruit form, and storage organ identity.

## 3. Molecular Regulatory Network of KNOX1 in Horticultural Crops

**Figure 1:**
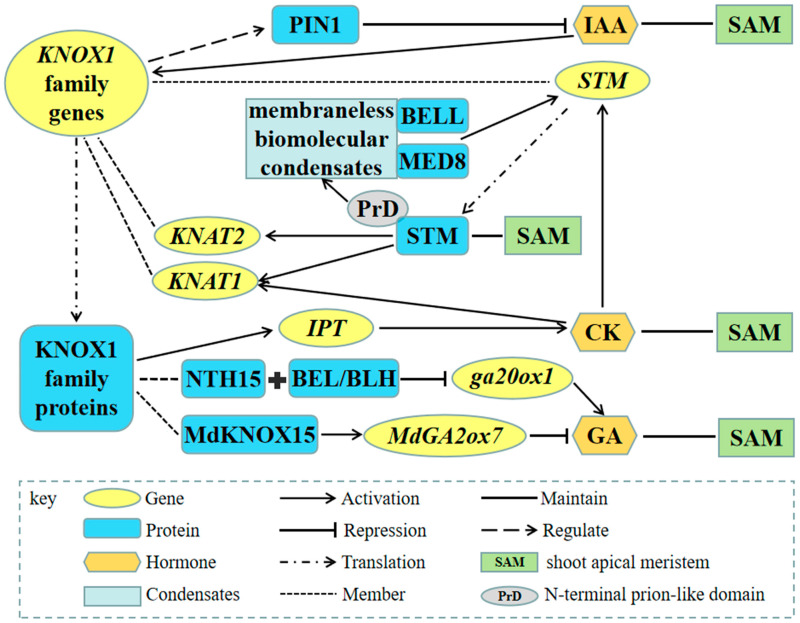
Molecular regulatory network of *KNOX1* genes in horticultural crops and the model organism Arabidopsis [28,31,32,33,34,35]. The KNOX1 N-terminal domain mediates the formation of protein heterodimers (KNOX1–BELL complexes) that function as key transcriptional regulators. These complexes bind to target gene promoters containing the TGAC core sequence, thereby activating or repressing downstream gene expression. Through this dual regulatory capacity, KNOX1 proteins modulate phytohormone metabolism and signaling cascades, including CK, GA, and IAA pathways, to maintain SAM stem cell identity and regulate organogenesis. Arrows indicate activation; blunt lines indicate suppression.

Studies have established that *KNOX1* genes precisely regulate the development of horticultural crops by modulating phytohormone metabolism and signaling, including CK, GA, and IAA. The *KNOX1* regulatory architecture spans core biosynthetic pathways, hormone crosstalk, upstream and downstream molecular interactions, and epigenetically governed spatiotemporal programs that integrate conserved and adaptive regulatory modules (Figure 1). This multi-layered framework positions *KNOX1* as a central integrator that coordinates hormonal inputs into developmental outputs across horticultural species.

### 3.1. Core Regulatory Pathway

*STM* stands as the foundational member of the *KNOX1* gene family. A landmark 1996 study in *Arabidopsis thaliana* convincingly established STM as an indispensable regulator of shoot apical meristem (SAM) initiation and homeostatic maintenance and uncovered the now-classic STM–KNOX1 core positive feedback regulatory loop. Recent genome-wide binding analysis via ChIP-seq has further verified that the STM protein can directly bind to the promoter regions of other *KNOX1* family genes, such as *KNAT1* and *KNAT2,* and activate their transcription. The proteins encoded by these family members in turn maintain and enhance *STM* expression, forming a self-reinforcing bistable regulatory circuit [31]. This feedback module is largely conserved across cucumber, tomato, apple, and other horticultural crops, yet interspecies divergence in promoter binding affinity alters meristem activity output [10,28]. Recent investigations have dramatically expanded our mechanistic understanding of this loop, revealing that the N-terminal prion-like domain (PrD) of the STM protein drives liquid–liquid phase separation, generating membraneless biomolecular condensates within the nucleus. These condensates co-localize and stably assemble with BELL family interaction partners and the Mediator complex subunit MED8, substantially potentiating the transcriptional activation capacity of *STM*. This phase-separation-mediated mechanism simultaneously activates both *STM* itself and its downstream target genes, reinforcing the integrity of the STM–KNOX1 pathway and ensuring the precise, robust maintenance of SAM stem cell activity [32]. The phase separation function of *KNOX1* remains underexplored in most horticultural crops, representing a critical research gap. These findings position STM not merely as a conventional transcription factor but as a phase-separation-driven master regulator whose biophysical properties are intrinsically linked to its developmental function.

### 3.2. Hormone Cross-Regulation

KNOX1 proteins exemplified by tobacco NTH15 form specific heterodimers with BEL/BLH family partners that bind directly to the promoter of the critical GA biosynthetic gene *ga20ox1* and potently repress its transcription. This repression drives a precipitous decline in bioactive GA levels, culminating in the characteristic dwarf and compact plant architecture associated with KNOX1 overexpression [33]. Similarly, *MdKNOX15* in apple directly binds to and upregulates *MdGA2ox7*, which encodes a GA-deactivating enzyme, thereby constituting a second independent route for negative regulation of GA accumulation [34]. Notably, the GA synthesis-inhibiting module is conserved in *Arabidopsis* as an ancestral pathway shared by model plants and herbaceous horticultural plants. Meanwhile, the *KNOX1*-mediated GA catabolism pathway only functions as an auxiliary route in *Arabidopsis*, yet evolves into a dominant, woody crop-specific module in apple [28].

In parallel, KNOX1 family proteins directly activate transcription of *IPT* (isopentenyl transferase), the rate-limiting gene for CK biosynthesis, thereby stimulating endogenous CK production. In a reciprocal fashion, elevated CK levels feed back to upregulate *STM* and *KNAT1* expression, establishing a self-amplifying KNOX1–CK positive feedback loop [35]. The resulting accumulation of CK at the shoot apex suppresses SAM cell differentiation, promotes sustained cell division, and safeguards meristem stem cell potency. Conversely, high IAA concentrations at emerging leaf primordia impose a sharp boundary on *KNOX1* expression, thereby demarcating the spatial limits of organogenesis. KNOX1, in turn, modulates the local IAA concentration gradient by redirecting the polar localization of the auxin efflux carrier PIN1, thereby orchestrating the polarized establishment of lateral organs. This elegant antagonistic interplay between KNOX1 and IAA ensures the precise balancing of SAM stem cell maintenance against the orderly initiation of lateral organs, containing leaves, flowers, and fruits, a regulatory logic that underpins the morphogenesis of tomato compound leaves and cucumber floral organs in horticultural crops [28]. Collectively, both the KNOX1-CK feedback circuit and the KNOX1-IAA antagonistic module are evolutionarily conserved across *Arabidopsis* and horticultural crops; nevertheless, horticultural crops repurpose this conserved framework to develop distinctive organ structures that do not exist in *Arabidopsis*.

### 3.3. Upstream and Downstream Interaction Network

*KNOX1* expression and function are regulated by a multi-tiered network operating upstream and downstream that modulates the balance between meristem activity and cell differentiation, thereby coordinating diverse developmental traits in horticultural crops. Upstream, the AS1–AS2 heterodimer directly binds *KNOX1* promoters and recruits histone-modifying enzymes to silence transcription, regulating periclinal cell division and leaf morphogenesis [36]. At the post-transcriptional level, miRNA166 targets and cleaves HD-ZIP III transcripts, an upstream repressor of *KNOX1*, thereby indirectly derepressing *KNOX1* expression. KNOX1 positively regulates *CUC1*/*2* (*CUP-SHAPED COTYLEDON 1*/*2*), and their combined activity defines the boundary of cell proliferation versus differentiation. This regulatory circuit is buffered by miR164, which targets *CUC1*/*2* transcripts to confine *KNOX1* expression and coordinate meristem and organ development [37]. Collectively, the AS1-AS2 transcriptional repression module and the KNOX1-CUC1/2-miR164 cascade are highly conserved in *Arabidopsis* and horticultural crops; however, horticultural crops remodel the spatiotemporal expression patterns of these conserved pathways to produce unique leaf morphologies absent in *Arabidopsis* [28].

At the protein interaction level, KNOX1 must obligately heterodimerize with BLH family partners to achieve high-affinity DNA binding at target gene promoters and execute transcriptional regulatory functions, a requirement unambiguously confirmed by the tomato KNOX-BLH interaction systems [38]. The dependency on BLH cofactors for KNOX1 function is largely conserved from *Arabidopsis* to all well-characterized horticultural crops. The KNOX1–CUC1/2 regulatory module further participates in organ morphogenesis by delineating the boundary between proliferating and differentiating cell domains, governing leaf margin serration patterning in pineapple [8] and orchestrating the initiation and spatial arrangement of compound leaf leaflets in citrus [39], thereby demonstrating striking functional versatility across species.

In downstream target gene regulation, KNOX1 mediates multi-dimensional developmental control through several convergent mechanisms: direct transcriptional regulation of key lignin biosynthetic genes including *PAL*(*Phenylalanine ammonia lyase*), *C4H*(*cinnamate 4-hydroxylase*), and *4CL*(*4-coumarate*: *CoA ligase*) that determines the lignification degree of vascular tissues, exemplified by the mechanical strength of grape stems [40,41]; repression of chloroplast development regulators such as *GLK1/2*(*GOLDEN2-LIKE 1/2*), which maintains SAM cells in a low chloroplast differentiation state essential for preserving stem cell identity [42,43]; and simultaneous activation of cell cycle-promoting genes such as *CYCD3*(*Cyclin D3*) and *CDKA*(*Cyclin-Dependent Kinase A*) alongside inhibition of cell cycle inhibitors such as *KRP*(*Kip-Related Protein*), thereby driving rapid meristem cell division and furnishing the cellular foundation for organogenesis and morphogenesis [44]. All three downstream regulatory cascades have been extensively characterized in *Arabidopsis* and are retained across diverse horticultural crops; perennial woody fruit crops further expand the functional scope of *KNOX1* to modulate vascular mechanical traits unique to fruit tree vegetative growth [28].

### 3.4. Epigenetic and Spatiotemporal Regulation

*KNOX1* expression is subject to rigorous epigenetic regulation and precise spatiotemporal control. Compelling evidence demonstrates that CG methylation levels within *KNOX1* gene promoter regions serve as a molecular rheostat governing their expression patterns: hypomethylation permits meristem-specific *KNOX1* expression, whereas hypermethylation imposes a strict silencing barrier against ectopic expression in differentiated organs [45]. Trimethylation of histone H3 at lysine 27 (H3K27me3), a canonical epigenetic repression mark, constitutes an additional critical layer of spatiotemporal regulation. The AS1–AS2 complex recruits the histone modifier LHP1(LIKE HETEROCHROMATIN PROTEIN 1) to deposit H3K27me3 across *KNOX1* gene regions, thereby enforcing transcriptional silencing and achieving exquisitely precise epigenetic control of *KNOX1* expression [46,47].

In a breakthrough application of spatiotemporal transcriptomics, cucumber *KNAT2-like1*, a *KNOX1* family member, was found to be sharply enriched in the floral intercalary meristem (FIM), where it drives rapid receptacle cell proliferation by modulating the CRC–ER regulatory module, ultimately determining the formation of the cucumber inferior ovary [10]. In contrast, *Arabidopsis* has no KNOX1-dependent pathway to control inferior ovary development. While horticultural crops, including tomato, apple, and citrus, maintain the completely conserved epigenetic repression mechanism of *KNOX1*, no evidence shows that they rely on *KNOX1* to form inferior ovaries according to existing studies. Only cucurbit crops produce differentiated functions via this conserved regulatory network and develop unique inferior ovary traits specific to this plant group.

## 4. KNOX1 Regulates Key Traits of Horticultural Crops

As a master regulatory transcription factor family governing plant development, *KNOX1* genes exert multifaceted and indispensable control over the formation of key agronomic and ornamental traits in horticultural crops by integrating hormonal signaling cascades, sustaining meristem activity, and directing cell fate determination. Their molecular mechanisms and breeding application value have been robustly and extensively validated across a diverse array of horticultural species, firmly establishing *KNOX1* as a cornerstone of both fundamental developmental biology and translational crop improvement.

### 4.1. Plant Architecture and Branching Regulation

Plant architecture constitutes a core agronomic trait that fundamentally governs the adaptation of horticultural crops to diverse cultivation regimes and their overall production efficiency. *KNOX1* genes have emerged as the principal regulatory determinants of plant architecture by precisely modulating meristem activity and hormonal equilibrium. In *Arabidopsis thaliana*, the core *KNOX1* family members *STM* and *KNAT1* orchestrate apical meristem maintenance, axillary bud differentiation, and stem development, collectively serving as the master regulatory framework for plant architecture establishment, a function that has been rigorously conserved across higher plants [48,49]. In horticultural crop systems, this regulatory logic has been leveraged with remarkable specificity. In lettuce (*Lactuca sativa* L.), the LsKN1–LsOFP6 protein interaction module activates the *LsKN1* allele *LsKN1TP* to suppress bolting through modulation of the GA signaling pathway, with LsOFP6 binding directly to LsKN1TP to safeguard GA biosynthesis and responsiveness [50]. The citrus *CclKNOX3* is specifically expressed in stems, lateral buds, and shoot tips; its overexpression induces intensive differentiation of the shoot apical meristem and leaf curling, and it coordinately regulates citrus shoot development and plant architecture formation with *CclKNOX5* [51]. Among the 19 *PbKNOX* genes in pear, *KNOX1* subfamily members are markedly upregulated in vigorous cultivars and conspicuously downregulated in dwarf mutants and dwarf hybrid progeny, with expression levels exhibiting a strong positive correlation with plant height, implicating *KNOX1* as a direct regulator of internode elongation and overall stature [52]. Genome-wide identification revealed that Class I *KNOX* genes, including *IbKNOX3* and *IbKNOX8*, are significantly upregulated in initial storage roots compared to fibrous roots in sweet potato, indicating their conserved roles in storage root formation [53]. Collectively, these findings across diverse horticultural species unambiguously establish that *KNOX1* genes achieve precise, multi-dimensional control of plant architecture. Relative to the conserved *STM*/*KNAT1*-dependent framework governing meristem maintenance and branching in *Arabidopsis*, KNOX1 pathways in horticultural crops retain deeply conserved core logic while evolving prominent lineage-specific innovations. Novel protein partners (*LsOFP6* in lettuce), paralog subfunctionalization (*CclKNOX3/5* in citrus), and trait-associated expression divergence (*KNOX1* expression linked to plant height in pear) drive rewiring of *KNOX1* regulatory circuits for specialized architectures, forming a direct link between conserved developmental mechanisms and precision crop improvement.

### 4.2. Leaf Shape and Ornamental Traits

Leaf shape stands as a critical determinant of both ornamental value and commercial quality in horticultural crops. *KNOX1* genes dominate the diversification of leaf morphology by integrating hormonal signals, governing leaf dorsiventrality, and sustaining meristematic potential, establishing themselves as the principal genetic drivers of leaf form innovation. In *Arabidopsis thaliana*, the *KNOX1* family members *KNAT1*, *KNAT2*, and *KNAT6* orchestrate leaf morphogenesis by suppressing deterministic cell differentiation, with their activity precisely antagonized by the AS1/RS2 repressor complex [36,54]. This deeply conserved regulatory framework has undergone extensive functional diversification in horticultural crops, endowing *KNOX1* genes with the capacity to mediate an extraordinary spectrum of leaf shape variation. In lettuce, upregulated expression of *LsKN1* drives a dramatic transformation from pinnately divided to palmately lobed leaves by simultaneously stimulating IAA biosynthesis, suppressing GA production, and downregulating the leaf dorsiventrality gene *LsAS1* [55]. Heterologous overexpression of *LtKNOX1* from *Lilium tsingtauense* induces malformed phenotypes, including leaf wrinkling, curling and crimping in *Nicotiana benthamiana*, and regulates leaf shape development in ornamental garden flowers [56]. Three *KNOX1* genes, *CmKNAT1*, *CmKNAT6*, and *CmSTM*, cloned from chrysanthemum, are all targeted to the nucleus, and their overexpression in tobacco and *Arabidopsis* produces a striking wrinkled leaf phenotype attributable to tissue hyperplasia driven by excessive and uncontrolled leaf cell division [57]. An InDel polymorphism in citrus *CiKNAT6* determines the ternate compound leaf trait; knockout of this gene increases leaf area, whereas ectopic overexpression induces leaf curling and reduces leaf area, and *CiKNAT6* participates in regulating compound leaf morphogenesis in citrus [58]. In the tea plant, *CsKNOX1* is sharply upregulated during early bud development, implicating it in the regulation of adventitious bud initiation, leaf lobe patterning, and curling morphology [59]. While the core KNOX1-AS1/RS2 antagonistic module that suppresses cell differentiation is deeply conserved across *Arabidopsis* and horticultural species, its downstream functional outputs have diversified through recalibrated hormonal crosstalk (*LsKN1*-mediated IAA/GA balance in lettuce), regulatory and coding sequence variation (*CiKNAT6* InDel in citrus), and paralog neofunctionalization (*CmKNAT1*/*6*/*STM* in chrysanthemum). This has yielded a far broader range of leaf morphologies than the repertoire of Arabidopsis, underscoring both the robustness of the ancestral leaf developmental program and the evolutionary plasticity that positions *KNOX1* as a prime target for precision foliage trait improvement.

### 4.3. Flower Development and Sex Determination

Flower development and sex differentiation constitute fundamental determinants of reproductive efficiency and yield potential in horticultural crops. *KNOX1* genes occupy a central position throughout the entire flower developmental program and in the maintenance of sexual identity, exerting decisive control over floral organ formation, hormonal homeostasis, and receptacle morphogenesis. In *Arabidopsis thaliana*, *KNOX1* family members participate in floral meristem maintenance and floral organ differentiation, with *KNAT1* and *KNAT2* orchestrating inflorescence architecture and floral organ patterning, providing a deeply conserved mechanistic reference for deciphering flower development in horticultural species [60]. Tomato *TKN3* is specifically expressed in the ovary wall/pericarp after anthesis; its mutation leads to pleiotropic phenotypes including enlarged floral organs and fruit developmental defects, and it participates in regulating post-anthesis fruit development through the auxin and GA pathways [61]. In apple, *MdKNOX15* governs flower induction and floral bud development, with its expression negatively regulated by the upstream growth regulator MdGRF, thereby fine-tuning floral organ formation [62]. In cucumber, *KNAT2* acts as a master determinant of female flower identity by orchestrating receptacle development, actively suppressing masculinization and bisexual flower differentiation, and directing inferior ovary formation [10]. *PmKNAT2/6-a* in Japanese apricot (*Prunus mume*) is dually repressed by PmAGL24 through two pathways: direct transcriptional repression via promoter binding, and indirect repression by recruiting PmLHP1 to deposit the repressive H3K27me3 mark. Elevated *PmKNAT2/6*-a expression triggers multi-pistil flower formation, and the gene acts as a key regulator of pistil number determination during floral development [63]. Taken together, *KNOX1* retains a deeply conserved function in floral meristem maintenance and organogenesis from *Arabidopsis* to horticultural crops, yet its functional scope has expanded substantially to include sex determination, pistil number specification, and post-anthesis fruit development. Most significantly, in cucurbit crops such as cucumber, their synergistic governance of inferior ovary formation and unisexual flower development constitutes a breakthrough molecular target poised to revolutionize sex-controlled breeding strategies.

### 4.4. Fruit Development and Quality

Fruit development and quality formation represent the ultimate expression of commercial value in horticultural crops. *KNOX1* genes are deeply embedded in the regulation of core fruit traits, relating size, shape, and maturation timing through coordinated modulation of hormone metabolism, chloroplast development, and cell differentiation programs. In *Arabidopsis thaliana*, the *KNOX1* family member *KNAT1* serves as a principal regulator of inflorescence architecture and silique development; its loss-of-function mutation produces shortened pedicels and internodes, recurved stem nodes, and consequent pleiotropic phenotypes including drooping flowers and siliques [49]. This extensively characterized regulatory paradigm in *Arabidopsis* provides a robust mechanistic foundation for dissecting fruit developmental programs in horticultural species. In maize, mutation of the *semaphore1* gene downregulates *KNOX1* expression, diminishes polar auxin transport, and severely compromises embryo, endosperm, and pollen development, revealing a critical KNOX1-dependent axis governing reproductive organogenesis [64]. In tomato, *TKN2* and *TKN4* function as upstream master regulators that activate the key chlorophyll biosynthesis and chloroplast development genes *SlGLK2* and *SlAPRR2-like*, establishing a chloroplast development gradient running from the calyx end to the fruit base that directly generates the characteristic uneven green pigmentation of immature fruits [43]. Tomato SlKD1, a master abscission regulator, disrupts the auxin-response gradient in the flower pedicel abscission zone through its interaction with the transcription factor SlGATA6, thereby inducing flower pedicel abscission to regulate tomato fruit setting and preharvest fruit drop [65]. In peach, *KNOPE1* modulates GA homeostasis by repressing GA biosynthetic gene expression during drupe development, thereby participating directly in mesocarp differentiation, a process central to fruit flesh quality determination [66]. In litchi, *LcKNAT1* is specifically expressed in the fruit abscission zone, and ectopic expression experiments demonstrate its capacity to suppress fruit drop by inhibiting ethylene biosynthetic gene expression, pointing to a promising target for reducing preharvest fruit loss [67]. In horticultural crops, *KNOX1* homologs have been co-opted to modulate an array of commercially critical fruit traits such as pigmentation, flesh texture and organ abscission, representing a substantial functional expansion beyond the conserved ancestral reproductive roles delineated in Arabidopsis. Representative examples include *TKN2*/*4*-driven chloroplast gradient formation in tomato fruit, *KNOPE1*-dependent mesocarp development in peach, and *LcKNAT1*-mediated inhibition of fruit abscission in litchi. This evolutionary plasticity underpins the role of KNOX1 as a core regulatory node for precision fruit quality breeding.

### 4.5. Abiotic Stress and Stress Resistance

Abiotic stress is the primary constraint on yield stability and quality in horticultural crops. *KNOX1* genes are promising targets for stress-resistance breeding that act through stress-signaling cascades, hormonal homeostasis, ROS scavenging, and stress-adaptive architectural remodeling. In *Arabidopsis thaliana*, ABA signaling induces *MYB96*, which directly upregulates *STM* to enhance drought tolerance [68]. Concurrently, the STM protein harbors a PrD that drives the formation of nuclear biomolecular condensates, substantially bolstering salt stress resilience [32]. These foundational investigations in *Arabidopsis* have established the mechanistic framework guiding stress response research in horticultural species. In radish, *RsKNAT3* enhances thermotolerance by directly activating *RsDREB2A* expression and scavenging ROS, while *RsKNAT1* antagonistically counteracts *RsKNAT3* function, suppressing *RsDREB2A* regulation under heat stress, revealing a sophisticated intraspecies KNOX1 regulatory circuit that dynamically calibrates stress responses [69]. In soybean, the expression of *GmKNOX1* genes responds to salt and dehydration stresses and participates in regulating abiotic stress resistance [70]. In poplar, *PagKNAT2/6b* suppresses GA biosynthesis to inhibit cell elongation and expansion, resulting in shortened internodes, reduced leaf area, and significantly enhanced drought resistance, demonstrating that *KNOX1*-mediated architectural remodeling constitutes a potent drought avoidance strategy [71]. *KNOX1* in *Vitis amurensis* exhibits tissue-specific expression, among which *VaKNOX6* and *VaKNOX7* are significantly upregulated under cold stress and participate in cold response regulation in Vitis amurensis [22]. In upland cotton, *GhKNOX10* and *GhKNOX14* serve as indispensable determinants of salt tolerance. Their silencing via VIGS precipitates a dramatic decline in seedling survival under salt stress, accompanied by markedly elevated ROS accumulation, unambiguously confirming their essential role in oxidative stress defense [72]. Collectively, these findings across diverse crop species establish that *KNOX1* genes possess extensive and versatile regulatory capacity in mediating abiotic stress responses, including high temperature, cold, drought, and salinity, and have evolved species-specific stress resistance networks through functional diversification and synergistic regulatory action, positioning them as high-priority targets for next-generation stress-resilient crop breeding.

**Figure 2:**
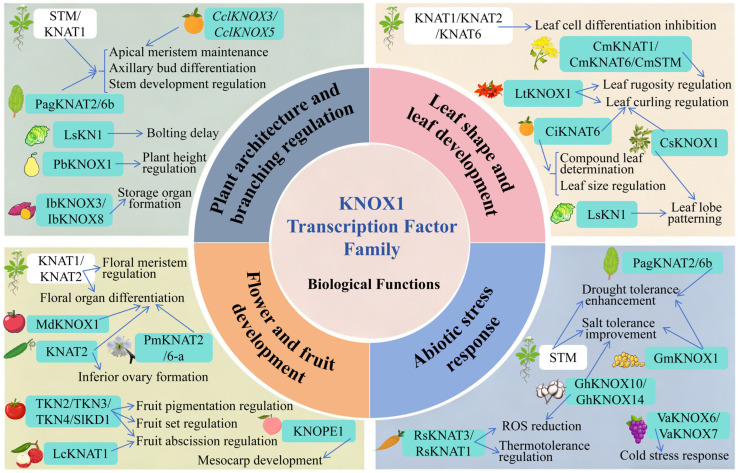
Schematic illustration of the biological functions of the KNOX1 transcription factor family in the model plant *Arabidopsis thaliana* and horticultural crops. As a core developmental regulatory hub, the KNOX1 transcription factor family integrates phytohormone and environmental stress signals to participate in four major biological processes: plant architecture and branching regulation, leaf shape and leaf development, flower and fruit development, and abiotic stress response. Each module presents representative functional genes of the *KNOX1* family from the model plant *Arabidopsis thaliana* and diverse horticultural crops. Arrows indicate the regulatory direction of genes toward corresponding phenotypic outputs.

In summary, *KNOX1* genes as master developmental transcription factors are deeply and pervasively embedded in the regulation of virtually every major agronomic and ornamental trait in horticultural crops, including plant architecture, leaf morphology, flower development, fruit quality, and abiotic stress resilience, through their capacity to integrate hormonal signaling cascades and sustain meristem activity. Their extraordinary functional breadth and mechanistic versatility position them as premier multi-functional targets for molecular breeding, offering substantial theoretical and practical support for next-generation crop improvement. To provide a comprehensive and intuitive visualization of the functional diversification patterns and deeply conserved regulatory principles governing the *KNOX1* gene family across *Arabidopsis* thaliana and diverse horticultural species, this review systematically compiles the gene nomenclature, species origin, and characterized biological functions of key family members (Table 1), and further presents a schematic diagram that systematically outlines the biological functions of the *KNOX1* family in four core biological processes (Figure 2). These curated data not only robustly corroborate the molecular mechanism conclusions delineated throughout this review but also furnish an authoritative reference foundation to catalyze the future identification, functional characterization, and breeding exploitation of novel *KNOX1* genes in horticultural crops.

## 5. Summary and Prospects

Since the inception of *KNOX1* gene family research, the associated technological landscape has gradually matured into a comprehensive pipeline spanning the full continuum from gene discovery to breeding deployment. Gene cloning and family identification have been propelled by genome-wide screening, homology-based cloning, and rigorous phylogenetic analysis, collectively elucidating the evolutionary trajectory and functional diversification mechanisms of KNOX proteins, furnishing a robust theoretical foundation for deciphering the functional differentiation principles governing *KNOX1* genes [20]. At the expression analysis level, researchers have synergistically integrated qPCR, transcriptomics, and in situ hybridization technologies. The application of spatial transcriptomics has provided a higher-resolution approach for dissecting cellular heterogeneity within the shoot apical meristem [73]. In functional validation, CRISPR/Cas9 and its derived tools have become the mainstream reverse genetics approach. Recent studies have summarized novel genome-editing strategies to manipulate apical meristem activity by tuning the KNOX and CLAVATA/WUSCHEL (CLV/WUS) negative feedback loop, including precise regulation via prime editing and epigenetic modification, which offer more accurate tools for plant architecture improvement in horticultural crops such as potato [74,75]. To circumvent the pervasive challenge of gene redundancy, multiplexed editing strategies have been widely adopted. In mechanistic interaction studies, yeast two-hybrid, dual-luciferase reporter, BiFC, and ChIP-seq assays have been systematically employed to reconstruct multi-layered regulatory networks [76]. At the breeding application frontier, as a molecular integrator of cell elongation and heat-stress response, *KNOX1* genes open a novel avenue for synergistic breeding of high yield and thermotolerance in horticultural crops [77].

Building upon the maturity of this integrated technological framework, the *KNOX1* family has demonstrated multi-dimensional breeding value, particularly in shaping plant architecture, fruit quality, and stress resilience, enabling a rapid transition from fundamental discovery to field-scale validation. In plant architecture improvement, precise modulation of *KNOX1* family gene expression levels offers a direct and effective route to architecturally optimized crop ideotypes. Quality breeding represents one of the most actively pursued frontiers of *KNOX1* application research. Through coordinated regulation of meristem activity and cell division dynamics, *KNOX1* genes exert direct influence on fruit nutritional composition and fruit set characteristics. Notably, while the KNOX-GLK regulatory module governing cell differentiation state is evolutionarily conserved across land plants, in tomato fleshy fruits, *KNOX1* members *TKN2* and *TKN4* have been co-opted to establish an axial chloroplast development gradient via direct transcriptional activation of the *SlGLK2* gene. This lineage-specific regulatory pattern governs chlorophyll accumulation in immature fruits and ultimately shapes the nutritional and flavor quality of ripe fruits [43]. Furthermore, recent studies have further demonstrated that tomato TKN3 controls tomato fruit size by regulating pericarp cell expansion, and its mutation leads to a dramatic reduction in fruit weight, providing new strategies for fruit size regulation under protected cultivation [61]. In stress resistance breeding, *KNOX1* genes exhibit substantial and growing application potential. Accumulating evidence confirms that the *KNOX1* family can confer robust tolerance to multiple abiotic stresses, including drought, salinity, high temperature, and high humidity through diverse mechanistic pathways encompassing ABA signaling modulation, reactive oxygen species scavenging, and stress-adaptive plant architecture remodeling [69,70,71,72]. Most significantly, gene-edited lines targeting potato *StKNOX1* have advanced to field trial evaluation [75], marking a pivotal milestone in the translational trajectory of *KNOX1* research from laboratory bench to open-field validation. This landmark achievement furnishes a compelling end-to-end proof of concept, spanning gene discovery through functional characterization to varietal improvement, and establishes a replicable blueprint for accelerating molecular breeding innovation in horticultural crops.

Despite the substantial progress achieved, the translation of *KNOX1* functional knowledge from fundamental mechanistic insights to industrial-scale breeding applications continues to face formidable bottlenecks and challenges rooted in persistent limitations of both research depth and breadth. At the molecular mechanism level, although KNOX1 downstream targets, interactions with BELL/BLH partners, and hormonal crosstalk involving GA, CK, and IAA have been preliminarily characterized, a coherent, integrative regulatory framework that spans multiple crops and coordinates diverse traits remains conspicuously absent. Current understanding remains largely confined to isolated, species-specific regulatory fragments rather than unified, translatable mechanistic models, and substantial gaps remain, particularly in the reconstruction of regulatory networks at the single-cell level and integrated multi-omics analyses [78,79]. At the species-difference level, a fundamental tension between functional conservation and species-specific divergence remains an important challenge. Conclusions derived from model plant studies resist direct extrapolation to perennial woody horticultural species such as citrus, pear, and litchi; compounding this problem, the notoriously low genetic transformation efficiency and protracted regeneration cycles of woody crops severely impede functional validation and mechanistic dissection [80]. At the species-coverage level, *KNOX* gene research remains disproportionately anchored to model plants, with horticultural crop studies remaining sporadically reported. Investigation is overwhelmingly concentrated on a narrow subset of fruit and vegetable species, principally pineapple, potato, and peach, while functional characterization of *KNOX1* in the vast majority of ornamental flowers and medicinal horticultural plants remains largely unexplored. At the breeding application level, constitutive overexpression of KNOX1 frequently triggers deleterious pleiotropic effects, including distorted plant architecture and disrupted flowering, rendering it unsuitable for direct varietal deployment. Furthermore, the overwhelming majority of horticultural *KNOX1* studies remain confined to controlled indoor or greenhouse environments, leaving the critical validation of multi-gene interaction networks and gene-by-environment (G×E) effects under open-field conditions extremely limited. Crop performance under natural stress regimes and functional crosstalk with agronomically relevant genes remain largely uncharacterized [78], collectively constituting a major barrier to breeding translation that severely constrains the conversion of laboratory discoveries into deployable field varieties.

Building upon current advances and remaining knowledge gaps, future research on *KNOX1* in horticultural crops should focus on several complementary priorities spanning mechanistic dissection, functional validation, precision gene editing, and field-based breeding applications. At the molecular mechanism level, leveraging cutting-edge pan-genome, single-cell, and spatial transcriptomics technologies will be essential to construct comprehensive spatiotemporal expression atlases of *KNOX1* across key organs, systematically identify its target gene repertoire and protein interaction partners, and elucidate the cross-regulatory interplay between epigenetic modifications and GA/CK/IAA signaling pathways, thereby helping to bridge critical knowledge gaps surrounding KNOX1 regulatory networks under natural field conditions. At the translational breeding level, deploying precision gene editing platforms such as CRISPR/Cas9 for tissue-specific and promoter-engineered modulation of *KNOX1* expression will enable the targeted optimization of plant architecture, fruit development, and stress resilience traits. Coupling these edited lines with rigorous multi-environment field trials to evaluate G×E interactions, trait stability, and agronomic performance across diverse agroecological zones will be indispensable for dismantling the translational barrier separating laboratory proof-of-concept from deployable commercial varieties. At the evolutionary developmental level, systematic cross-species comparative analyses centered on hallmark horticultural traits, including inferior ovary formation, compound leaf patterning, and tuber morphogenesis, together with functional validation in underexplored horticultural species, will clarify the conserved and divergent regulatory logic of *KNOX1* in organ morphological evolution, furnishing a molecular framework for understanding plant morphological diversification and guiding future trait-oriented improvement in horticultural crops.

## Figures and Tables

**Table 1:** Summary of biological functions of the *KNOX1* gene family in model plant *Arabidopsis thaliana* and horticultural crops.

Functional Pathway	Gene Name	Species	Functional Characteristics	References
Plant architecture and branching regulation	*STM/KNAT1*	*Arabidopsis thaliana*	Maintains apical meristem activity, regulates axillary bud differentiation, and stem development.	[48,49]
	*LsKN1*	*Lactuca sativa* L.	Regulates the GA pathway to delay bolting and regulate leaf morphogenesis.	[50,55]
	*CclKNOX3/CclKNOX5*	*Citrus*	Is specifically expressed in stems and lateral buds; synergistically regulates shoot development and plant architecture.	[51]
	*PbKNOX1*	*Pyrus bretschneideri* Rehd.	Its expression level is positively correlated with plant height; regulates internode elongation.	[52]
	*PagKNAT2/6b*	*Populus alba* × *P. glandulosa*	Inhibits GA biosynthesis and regulates plant architecture establishment.	[71]
Leaf shape and leaf development	*KNAT1/KNAT2/KNAT6*	*Arabidopsis thaliana*	Inhibits deterministic differentiation of leaf cells and is negatively regulated by *AS1/RS2.*	[36,54]
	*LsKN1*	*Lactuca sativa* L.	Regulate hormones and other genes to change leaf shape.	[55]
	*LtKNOX1*	*Lilium tsingtauense*	Heterologous overexpression induces leaf wrinkling, curling, and malformation.	[56]
	*CmKNAT1/CmKNAT6/CmSTM*	*Chrysanthemum* × *morifolium*	Wrinkled leaf phenotype induced by heterologous overexpression.	[57]
	*CiKNAT6*	*Citrus*	An InDel polymorphism determines the ternate compound leaf trait; regulates leaf size.	[58]
	*CsKNOX1*	*Camellia sinensis* (L.) O. Kuntze	Regulates bud development and leaf lobes, curly leaf shapes.	[59]
Flower and fruit development	*KNAT1/KNAT2*	*Arabidopsis thaliana*	Regulates the floral meristem, inflorescence structure, and floral organ differentiation, which affect silique development.	[49,60]
	*TKN2/TKN3/TKN4*	*Solanum lycopersicum* L.	Regulates post-anthesis fruit development (TKN3, auxin/GA pathways) and chloroplast/pigmentation patterning (TKN2/TKN4).	[43,61]
	*SlKD1*	*Solanum lycopersicum* L.	Regulates fruit set and preharvest abscission by interacting with SlGATA6 to disrupt auxin gradients in the pedicel abscission zone.	[65]
	*MdKNOX1*	*Malus domestica*	Participate in flower induction and regulate floral organ formation.	[62]
	*KNAT2*	*Cucumis sativus* L.	Maintains female flower phenotype and regulates inferior ovary formation.	[10]
	*PmKNAT2/6-a*	*Prunus mume*	Induce multi-pistil flower formation and regulate floral development.	[63]
	*ZmKNOX1*	*Zea mays* L.	Reduces IAA polar transport and participates in embryo, endosperm, and pollen development.	[64]
	*KNOPE1*	*Prunus persica* L. Batsch	Regulate GA homeostasis and participate in mesocarp development.	[66]
	*LcKNAT1*	*Litchi chinensis* Sonn.	Inhibits ethylene synthesis and delays fruit abscission.	[67]
Storage organ development	*IbKNOX3/IbKNOX8*	*Ipomoea batatas*	Significantly upregulated in initial storage roots, regulating tuber formation.	[53]
Abiotic stress response	*STM*	*Arabidopsis thaliana*	Is induced by ABA to enhance drought tolerance, forming nuclear condensates to improve salt tolerance.	[32,68]
	*RsKNAT3/RsKNAT1*	*Raphanus sativus* L.	*RsKNAT3* enhances heat tolerance, and the two antagonize this regulatory effect.	[69]
	*GmKNOX1*	*Glycine max* (L.) Merr.	Responds to salt and dehydration stress, mediating abiotic stress resistance.	[70]
	*VaKNOX6/VaKNOX7*	*Vitis amurensis*	Are significantly upregulated under cold stress, regulating cold response.	[22]
	*PagKNAT2/6b*	*Populus alba* × *P. glandulosa*	Inhibits GA synthesis and enhances plant drought resistance via architectural remodeling.	[71]
	*GhKNOX10/GhKNOX14*	*Gossypium hirsutum* L.	Positively regulate salt tolerance and reduce ROS accumulation.	[72]

## Data Availability

No data was used for the research described in the article.
